# Survival Analysis of Patients with Interval Cancer Undergoing Gastric Cancer Screening by Endoscopy

**DOI:** 10.1371/journal.pone.0126796

**Published:** 2015-05-29

**Authors:** Chisato Hamashima, Michiko Shabana, Mikizo Okamoto, Yoneatsu Osaki, Takuji Kishimoto

**Affiliations:** 1 Cancer Screening Assessment and Management Division, Research Center for Cancer Prevention and Screening, National Cancer Center, Tokyo, Japan; 2 Department of Internal Medicine, San-in Rosai Hospital, Yonago, Tottori Prefecture, Japan; 3 Division of Environmental and Preventive Medicine, Department of Social Medicine, Faculty of Medicine, Tottori University, Yonago, Tottori Prefecture, Japan; University Hospital Llandough, UNITED KINGDOM

## Abstract

**Aims:**

Interval cancer is a key factor that influences the effectiveness of a cancer screening program. To evaluate the impact of interval cancer on the effectiveness of endoscopic screening, the survival rates of patients with interval cancer were analyzed.

**Methods:**

We performed gastric cancer-specific and all-causes survival analyses of patients with screen-detected cancer and patients with interval cancer in the endoscopic screening group and radiographic screening group using the Kaplan-Meier method. Since the screening interval was 1 year, interval cancer was defined as gastric cancer detected within 1 year after a negative result. A Cox proportional hazards model was used to investigate the risk factors associated with gastric cancer-specific and all-causes death.

**Results:**

A total of 1,493 gastric cancer patients (endoscopic screening group: n = 347; radiographic screening group: n = 166; outpatient group: n = 980) were identified from the Tottori Cancer Registry from 2001 to 2008. The gastric cancer-specific survival rates were higher in the endoscopic screening group than in the radiographic screening group and the outpatients group. In the endoscopic screening group, the gastric cancer-specific survival rate of the patients with screen-detected cancer and the patients with interval cancer were nearly equal (P = 0.869). In the radiographic screening group, the gastric cancer-specific survival rate of the patients with screen-detected cancer was higher than that of the patients with interval cancer (P = 0.009). For gastric cancer-specific death, the hazard ratio of interval cancer in the endoscopic screening group was 0.216 for gastric cancer death (95%CI: 0.054-0.868) compared with the outpatient group.

**Conclusion:**

The survival rate and the risk of gastric cancer death among the patients with screen-detected cancer and patients with interval cancer were not significantly different in the annual endoscopic screening. These results suggest the potential of endoscopic screening in reducing mortality from gastric cancer.

## Introduction

Gastric cancer is the third leading cause of cancer death in both sexes worldwide, with its number reaching about 723,000 in 2012 [1]. Although half of the total number of gastric cancer has been reported in Eastern Asia, the burden of gastric cancer has also remained in Eastern and South Europe. In most countries, gastric cancer screening has not been commonly carried out, except in Korea and Japan which have performed gastric cancer screening as a national program [2, 3]. The Japanese screening program for gastric cancer is limited to upper gastrointestinal series using barium meal (i.e., radiographic screening), whereas the Korean screening program consists of both radiographic and endoscopic screenings. However, studies evaluating mortality reduction from gastric cancer by endoscopic screening remain limited [4, 5].

Mortality reduction from gastric cancer is a long-term effect of gastric cancer screening. On the other hand, evaluation of interval cancer can provide an early estimate of the impact of screening programs [6]. Interval cancer is defined as cases that are diagnosed after negative results of screening in the periods between routine and scheduled screenings [6]. The rate of interval cancer and the survival rate are directly affected the effectiveness of the cancer screening program. The sensitivity of endoscopic screening was previously calculated based on the rate of interval cancer using cancer registry data [7, 8]. On the other hand, there are only a few studies related to survival analysis of patients with gastric cancer detected by endoscopic screening [9, 10]. The survival of patients with interval cancer in endoscopic screening also remains unclear. To evaluate the impact of interval cancer on the effectiveness of endoscopic screening, the survival rates of patients with interval cancer were analyzed and compared with those of patients with screen-detected cancers between endoscopic and radiographic screenings based on the Tottori Cancer Registry in Japan.

## Methods

### Screening programs

The subjects of our study were selected from gastric cancer cases registered in 4 cities (i.e., Tottori, Yonago, Kurayoshi, and Sakaiminato) in Tottori Prefecture, Japan. Endoscopic screening has been conducted in Tottori, Yonago, and Sakaiminato since 2000 and in Kurayoshi since 2001. Gastric cancer screening is offered annually by local governments, and both radiography and endoscopy are used in these cities. All individuals aged 40 years and over can participate in the gastric cancer screening programs. There is no upper age limit for the target population for gastric cancer screening. Individuals can choose either endoscopy or radiography for gastric cancer screening based on their preference. Since the introduction of endoscopic screening, the participation rate in gastric cancer screening has increased, although the participation rate in gastric cancer screening involving both methods has remained at about 25% [11].

Physicians who can perform endoscopic screening were approved by the local committee for gastric cancer screening based on certain requirements [11]. Although endoscopic screening has been performed in clinical settings, the results have been evaluated based on monitor screen review by the local committee, including experienced endoscopists in each city.

### Target group

The subjects of our study were selected from gastric cancer cases registered in 4 cities (Tottori, Yonago, Kurayoshi, and Sakaiminato) in the Tottori Cancer Registry from 2001 to 2008. There were 2,066 potential subjects with gastric cancer in the 4 cities in Tottori Prefecture. Detailed information of all the potential cases was obtained from the local cancer registries, and the following cases were excluded: patients who 1) were more than 80 years old and less than 39 years old at the time of gastric cancer diagnosis, 2) had registry duplication, 3) lacked the diagnosis date for gastric cancer, or 4) had a diagnosis other than gastric cancer. The selected patients with gastric cancers were divided into 3 groups according to the detection process used in the participant list of gastric cancer screening from 2000 to 2006 in the 4 cities. Screening histories were investigated from the participant lists and matching was based on name, sex, and birthday. When there was no screening history, the patients were defined as belonging to the outpatient group.

The screening group was divided into patients with screen-detected cancer and patients with interval cancer based on the screening results. Patients with screen-detected cancer patients were identified after a positive result of gastric cancer screening. Since the screening interval of both endoscopic screening and radiographic screening was 1 year, interval cancer was defined as cancer detected within 1 year after a negative result on cancer screening.

### Follow-up

Follow-up was continued from the date of diagnosis to the date of death or up to December 31, 2011 based on the Tottori Cancer Registry. The mean follow-up period was 66.4 ± 38.6 months. Since the local cancer registry system did not collect the stages of all gastric cancers, we obtained detailed information from the database for gastric cancer screening of the Tottori Medical Association. However, information on gastric cancer patients who had never been screened was not available. Tumor location was recorded using the Japanese Classification of Gastric Carcinoma [12], in which the stomach is anatomically divided into 3 portions: upper, middle, and lower. Clinical stage was determined based on the Japanese Classification of Gastric Carcinoma [12]. Gastric cancers were also classified histologically into intestinal and diffuse types according to Lauren’s criteria [13].

### Statistical analysis

The characteristics of the target groups were compared using the chi-square test. Survival analysis was performed using the Kaplan-Meier method with the log-rank test. The obtained curves show the proportion of individuals alive over time starting at the time of cancer diagnosis. Gastric cancer-specific survival and all-causes survival rates were calculated. A Cox proportional hazards model was used to investigate the risk factors associated with gastric cancer death and all-causes death for the endoscopic and radiographic screening group. Analyses were carried out using STATA 13.0 (STATA, College Station, TX, USA). All test statistics were two tailed, and P values of < 0.05 were considered to indicate a statically significant difference.

### Ethics statement

This study used the data of the local cancer registry and the population lists of gastric cancer screening. These were not included in the informed consents for the collection of the screening results and health data. Based on the Japanese guideline for epidemiological studies developed by the national government, informed consent is not required for an observational study using secondary data without human materials [14]. Our study was survival analysis using the secondary data from the local cancer registry and the population lists of gastric cancer screening. Therefore, obtaining informed consent was waived in this study based on the Japanese guideline for epidemiological studies. This was confirmed by the Institutional Review Board of the National Cancer Center of Japan. Finally, this study was approved by the Institutional Review Board of the National Cancer Center of Japan on October 22, 2007.

## Results

**Fig 1:**
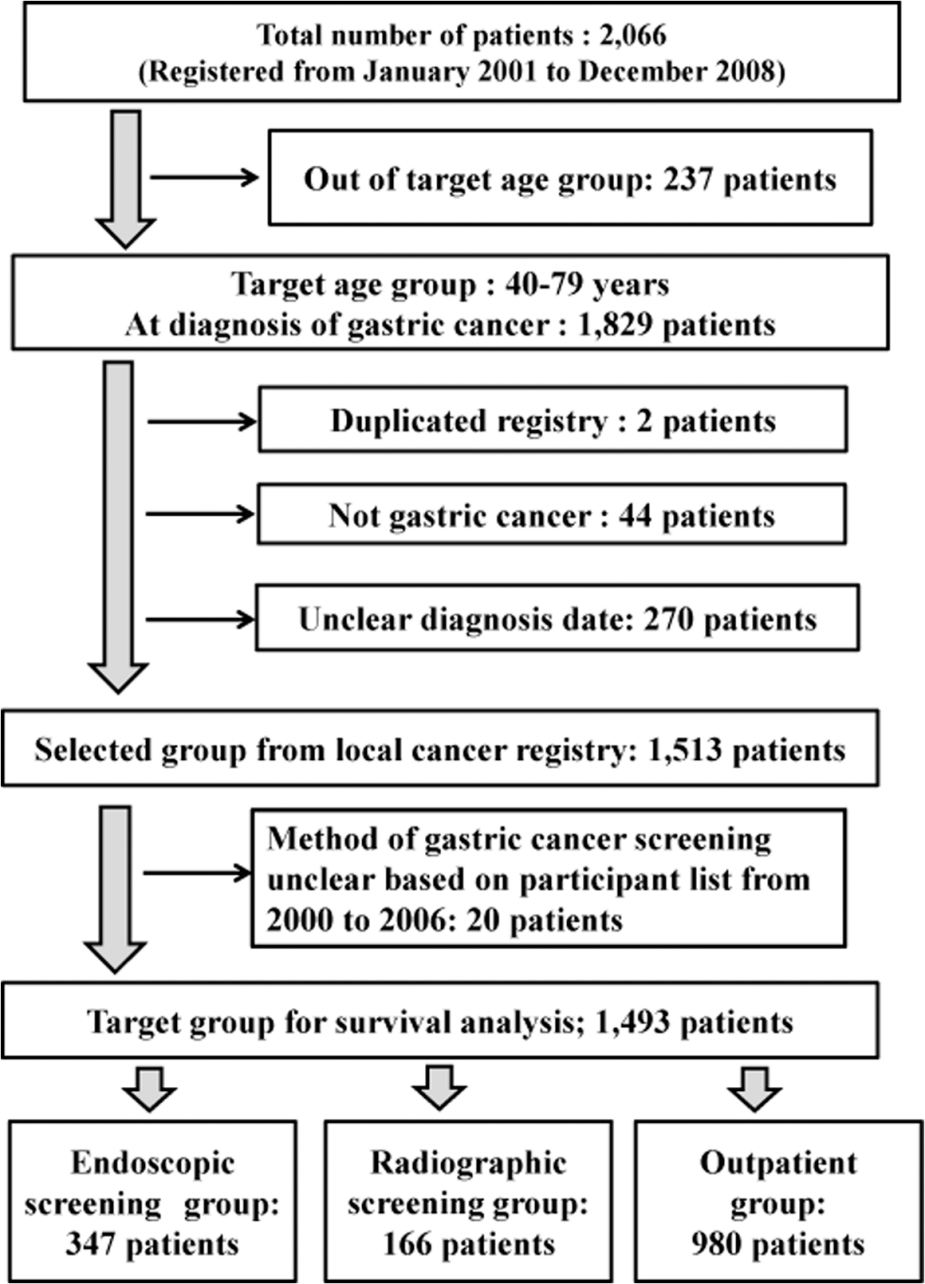
Flow-chart of the selection process for the target group. There were 2,066 potential subjects with gastric cancer in the 4 cities examined in Tottori Prefecture (i.e., Tottori, Yonago, Kurayoshi, and Sakaiminato). The following patients were excluded: those who 1) were over 80 years old and less than 39 years old at the time of gastric cancer diagnosis, 2) had registry duplication, 3) lacked the date for gastric cancer diagnosis, or 4) had a diagnosis other than gastric cancer. Two patients who had registry duplication, 44 patients who were not cases of gastric cancers, and 270 patients in whom the date of diagnosis was unclear were also excluded. From the local registry, 1,513 subjects were selected. Based on the participants list for gastric cancer from 2000 to 2006, 20 subjects whose screening methods were unclear were excluded. The remaining 1,493 subjects were divided into 3 groups according to the method of cancer detection: endoscopic screening group (n = 347), radiographic screening group (n = 166), and outpatient group (n = 980).

The procedure used for the selection of the target population is shown in **Fig 1**. A total of 2,066 subjects were selected from the Tottori Cancer Registry, of which 237 patients were not within the target age for the analysis. Most subjects who were excluded from the target group were more than 80 years old at the time of diagnosis, which was not the actual target for cancer screening. Two patients who had registry duplication, 44 patients who were not cases of gastric cancers, and 270 patients in whom the date of diagnosis was unclear were also excluded. From the list of participants with gastric cancer screening from 2000 to 2006, 20 patients whose screening methods were unclear were excluded. The remaining 1,493 patients were finally divided into 3 groups according to the cancer detection procedure as follows: endoscopic screening group (n = 347), radiographic screening group (n = 166), and outpatient group (n = 980; symptoms detected in outpatients). In the endoscopic screening group, the number of patients with screen-detected cancer was 324 and that of patients with interval cancer was 23. In the radiographic screening group, the number of patients with screen-detected cancer was 143 and that of patients with interval cancer was 23.

The results of the comparison of the basic characteristics of the endoscopic screening group, radiographic screening group, and outpatient group are shown in **Table 1**. The proportion of male patients was significantly higher than that of female patients in all groups. The age distribution was different between the 3 groups. Although more than 50% of the patients in the endoscopic and radiographic screening groups were 70 years and over, the proportion of the 70 years and over age group was lower in the outpatient group than in both the endoscopic and radiographic screening groups.

**Table 1:** Basic characteristics of the endoscopic screening group, radiographic screening group, and outpatient group.

	Endoscopic screening group		Radiographic screening group		Outpatient group		P-value
	Number of patients	(%)	Number of patients	(%)	Number of patients	(%)	
**Total number**	**347**		**166**		**980**		
**Age group**							
** 40–49 years**	**9**	**2.6**	**1**	**0.6**	**94**	**9.6**	**< 0.001**
** 50–59 years**	**25**	**7.2**	**15**	**9.0**	**254**	**25.9**	
** 60–69 years**	**122**	**35.2**	**46**	**27.7**	**273**	**27.9**	
** 70–79 years**	**191**	**55.0**	**104**	**62.7**	**359**	**36.6**	
**Sex**							
** Male**	**226**	**65.1**	**98**	**59.0**	**710**	**72.4**	**< 0.001**
** Female**	**121**	**34.9**	**68**	**41.0**	**270**	**27.6**	

In the outpatient group, detailed information could not be obtained from the Tottori Cancer Registry, and the clinical stage and location were unknown in more than 70% of the patients in the outpatient group. The characteristics of the patients with screen-detected cancer and patients with interval cancer were compared between the endoscopic and radiographic screening groups (**Table 2**). The proportion of stage I was approximately 50% among the screen-detected cancer in the endoscopic screening and radiographic screening groups. The clinical stage was unknown in most of the patients with interval cancer. The clinical stage distribution was not significantly different between the endoscopic screening group and the radiographic screening group (P = 0.415). The numbers of screen-detected cancer according to histological types using both screening methods were also not significantly different (P = 0.581).

**Table 2:** Comparison of the number of screen-detcted cancer and interval cancer in the endoscopic screening group and the radiographic screening group.

	Endoscopic screening group				Radiographic screening group			
	Screen-detected cancer		Interval cancer		Screen-detected cancer		Interval cancer	
	Number	(%)	Number	(%)	Number	(%)	Number	(%)
**Total number**	**324**		**23**		**143**		**23**	
**Sex**								
** Male**	**214**	**66.0**	**12**	**52.2**	**84**	**58.7**	**14**	**60.9**
** Female**	**110**	**34.0**	**11**	**47.8**	**59**	**41.3**	**9**	**39.1**
**Age group**								
** 40–49 years**	**9**	**2.8**	**0**	**0.0**	**1**	**0.7**	**0**	**0.0**
** 50–59 years**	**22**	**6.8**	**3**	**13.0**	**14**	**9.8**	**1**	**4.3**
** 60–69 years**	**116**	**35.8**	**6**	**26.1**	**36**	**25.2**	**10**	**43.5**
** 70–79 years**	**177**	**54.6**	**14**	**60.9**	**92**	**64.3**	**12**	**52.2**
**City**								
** Tottori**	**144**	**44.4**	**13**	**56.5**	**76**	**53.1**	**9**	**39.1**
** Yonago**	**137**	**42.3**	**5**	**21.7**	**46**	**32.2**	**8**	**34.8**
** Kurayoshi**	**9**	**2.8**	**0**	**0.0**	**9**	**6.3**	**4**	**17.4**
** Sakaiminato**	**34**	**10.5**	**5**	**21.7**	**12**	**8.4**	**2**	**8.7**
**Location**								
** U**	**68**	**21.0**	**0**	**0.0**	**27**	**18.9**	**6**	**26.1**
** M**	**148**	**45.7**	**11**	**47.8**	**74**	**51.7**	**8**	**34.8**
** L**	**103**	**31.8**	**11**	**47.8**	**37**	**25.9**	**7**	**30.4**
** Unknown**	**5**	**1.5**	**1**	**4.3**	**5**	**3.5**	**2**	**8.7**
**Stage**								
** I**	**181**	**55.9**	**2**	**8.7**	**77**	**53.8**	**1**	**4.3**
** Ⅱ**	**22**	**6.8**	**0**	**0.0**	**12**	**8.4**	**0**	**0.0**
** Ⅲ**	**24**	**7.4**	**0**	**0.0**	**8**	**5.6**	**0**	**0.0**
** Ⅳ**	**9**	**2.8**	**0**	**0.0**	**2**	**1.4**	**1**	**4.3**
** Unknown**	**88**	**27.2**	**21**	**91.3**	**44**	**30.8**	**21**	**91.3**
**Histology**								
** Intestinal type**	**226**	**69.8**	**18**	**78.3**	**94**	**65.7**	**13**	**56.5**
** Diffuse type**	**87**	**26.9**	**1**	**4.3**	**42**	**29.4**	**7**	**30.4**
** Others**	**2**	**0.6**	**1**	**4.3**	**2**	**1.4**	**0**	**0.0**
** Unknown**	**9**	**2.8**	**3**	**13.0**	**5**	**3.5**	**3**	**13.0**

U, Upper body; M, Middle body; L, Lower body

1) The location, histological type, and stage of all gastric cancers were studied. Tumor location was recorded using the Japanese Classification of Gastric Carcinoma, in which the stomach is anatomically divided into 3 portions, namely, upper, middle, and lower. [12]

2) Clinical stage was also used for determination of the clinical stage based on the Japanese Classification of Gastric Carcinoma [12].

3) Gastric cancers were also classified histologically into intestinal and diffuse types according to Lauren’s criteria [13].

**Fig 2:**
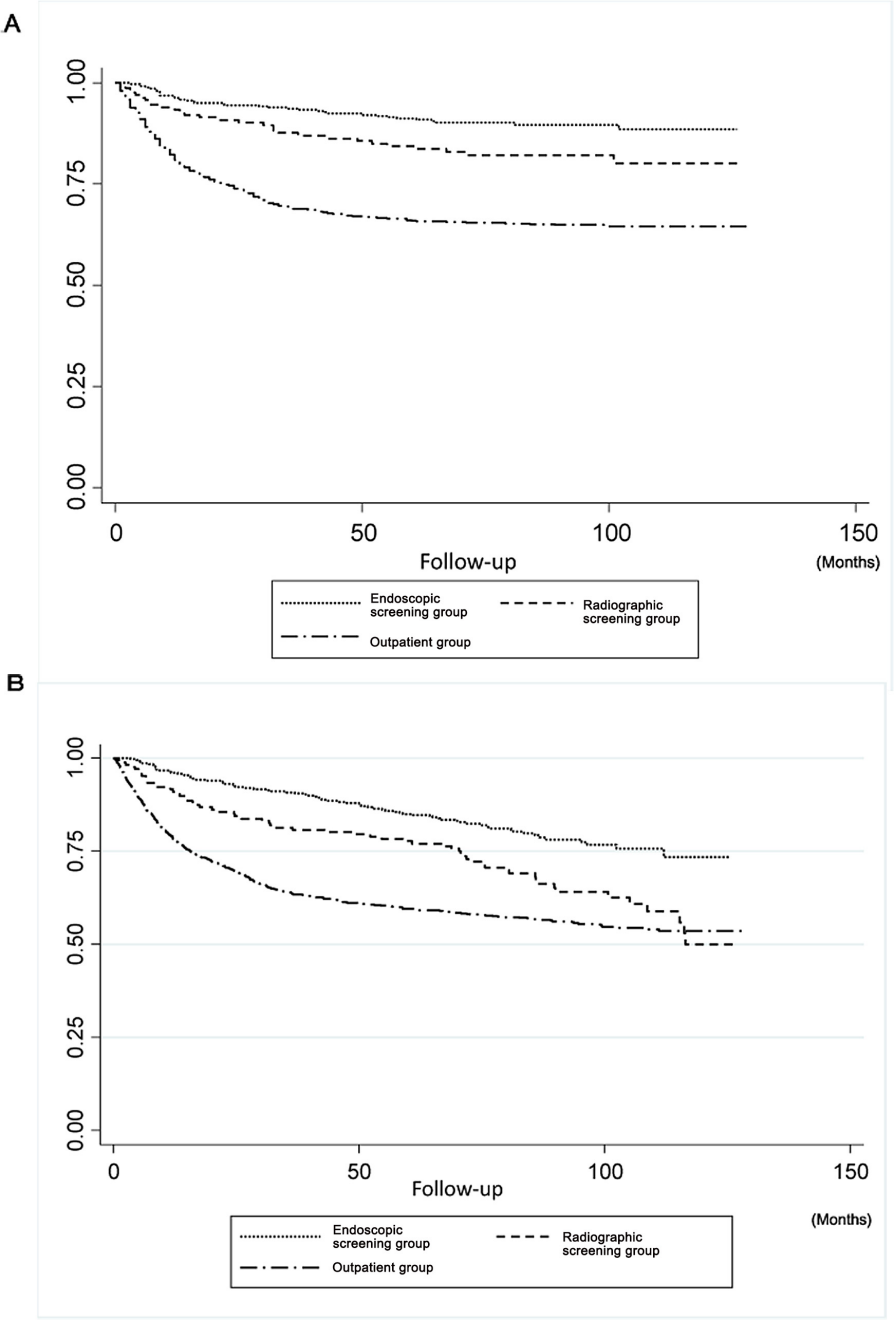
Survival analyses of gastric cancer patients classified under the endoscopic screening, radiographic screening, and outpatient groups. Of the 1,493 gastric cancer patients, 347 patients were classified under the endoscopic screening group, 166 patients under the radiographic screening group, and 980 patients under the outpatient group. **A.** Gastric cancer-specific survival rates of the 3 different groups. There were significant differences in the gastric cancer-specific survival rate between the endoscopic screening group and the outpatient group (P < 0.001), as well as between the radiographic screening group and the outpatient group (P < 0.001). The gastric cancer-specific survival rates of the patients in the endoscopic screening group was significantly higher than those of the patients in the radiographic group (P = 0.013). **B.** All-causes survival rates of the 3 different groups. There was a significant difference in the all-causes survival rate between the endoscopic screening group and the outpatient group (P < 0.001). The all-causes survival rate of the patients in the radiographic screening group was significantly higher than that of the patients in the outpatient group (P = 0.011). There was a significant difference in the all-causes survival rate between the endoscopic screening group and the radiographic group (P = 0.001).

The results of the Kaplan-Meier analysis of survival in patients with gastric cancer detected by screening and outpatients are shown in **Fig 2A**. The 5-year survival rates were 91.2 ± 1.5% (95%CI: 87.5–93.8) for the endoscopic screening group, 84.3 ± 2.9% (95%CI: 87.5–93.8) for the radiographic screening group, and 66.0 ± 1.6% (95%CI: 62.8–68.9) for the outpatient group. There were significant differences in the gastric cancer-specific survival rate between the endoscopic screening group and the outpatient group (P < 0.001), as well as between the radiographic screening group and the outpatient group (P < 0.001). The gastric cancer-specific rate was significantly higher in the patients in the endoscopic screening group than in the patients in the radiographic screening group (P = 0.013). There were significant differences in the all-causes survival rates between the endoscopic screening group and the outpatient group (P < 0.001) (**Fig 2B**). The all-causes survival rates of the radiographic screening group were also significantly higher than those of the outpatient group (P = 0.011). There were significant differences in the all-causes survival rates between the endoscopic screening group and the radiographic group (P = 0.001).

**Fig 3:**
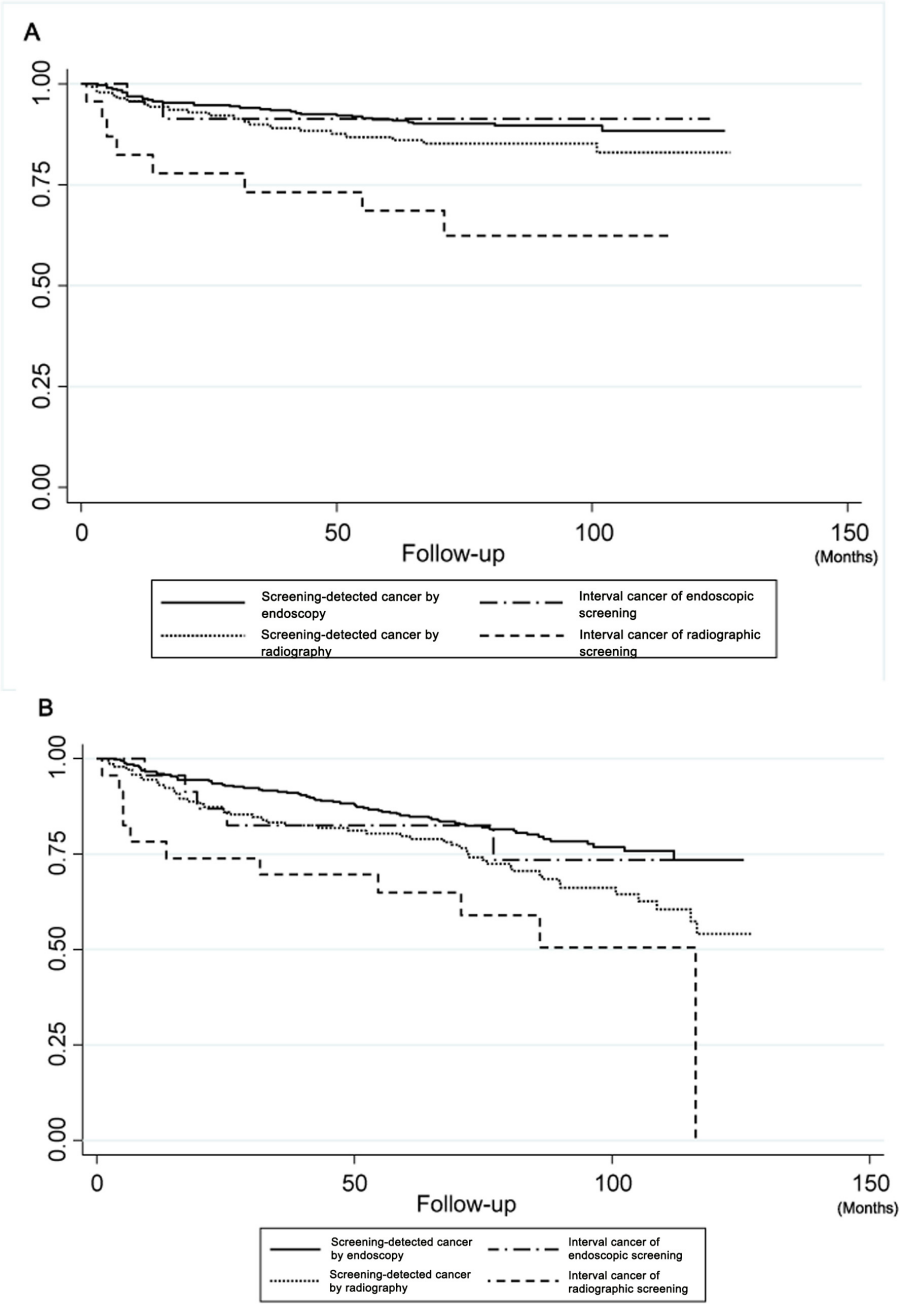
Survival analyses of patients with screen-detected cancer and patients with interval cancer in the endoscopic and radiographic screening groups. In the endoscopic screening group, there were 324 patients with screen-detected cancer and 23 patients with interval cancer. In the radiographic screening group, there were 143 patients with screen-detected cancer and 23 patients with interval cancer. **A.** Gastric cancer-specific survival rates of patients in the 4 different groups. In the endoscopic screening group, there was no significant difference in the gastric cancer-specific survival rates between the patients with screen-detected cancer and the patients with interval cancer (P = 0.869). The gastric cancer-specific survival rate was significantly higher in the patients with interval cancer in the endoscopic screening group than in the outpatient group (P = 0.018). In the radiographic screening group, there was a significant difference in the gastric cancer-specific survival rates between the patients with screen-detected cancer and the patients with interval cancer patients (P = 0.009). The gastric cancer-specific survival rate of the patients with interval cancer in the radiographic screening was not significantly different from that of the patients in the outpatient group (P = 0.961). **B.** All-causes cancer survival rates of patients with the 4 different groups. In the endoscopic screening group, there was no significant difference in the all-causes cancer survival rates between the patients with screen-detected cancer and the patients with interval cancer (P = 0.786). The all-causes survival rate of the patients with interval cancer in the endoscopic screening group was significantly higher than that of the patients in the outpatient group (P = 0.047). In the radiographic screening group, the all-causes cancer survival rate of the patients with screen-detected cancer was significantly higher than that of the patients with interval cancer (P = 0.045). The all-causes survival rate of the patients with interval cancer in the radiographic screening group was not significantly different from that of the patients in the outpatient group (P = 0.771).

The gastric cancer-specific survival rates of the patients with screen-detected cancer and patients with interval cancer in the screening groups are shown in **Fig 3A.** In the endoscopic screening group, the 5-year survival rate of the patients with screen-detected cancer was 91.9 ± 1.6% (95%CI: 87.5–93.8) and that of the patients with interval cancer was 91.3 ± 5.9% (95%CI: 69.5–97.8). In the radiographic screening group, the 5-year survival rate of the patients with screen-detected cancer was 86.8 ± 2.9% (95%CI: 79.9–91.5) and that of the patients with interval cancer was 68.7 ± 2.9% (95%CI: 45.2–83.7). In the endoscopic screening group, there were no significant differences in the gastric cancer-specific survival rates between the patients with screen-detected cancer and the patients with interval cancer (P = 0.869). The gastric cancer-specific survival rate was significantly higher in the patients with interval cancer in the endoscopic screening group than in the outpatient group (P = 0.018). In the radiographic screening group, there was a significant difference in the gastric cancer-specific survival rates between the patients with screen-detected cancer and the patients with interval cancer (P = 0.009). The gastric cancer-specific survival rate of the patients with interval cancer in the radiographic screening was not significantly different from that of the patients in the outpatient group (P = 0.961).

The all-causes survival rates of the patients with screen-detected cancer and patients with interval cancer patients in the screening groups are shown in **Fig 3B**. In the endoscopic screening group, there were no significant differences in the all-causes cancer survival rates between the patients with screen-detected cancer and the patients with interval cancer (P = 0.786). The all-causes survival rate of the patients with interval cancer in the endoscopic screening group was significantly higher than that of the patients in the outpatient group (P = 0.047). In the radiographic screening group, the all-causes survival rates of the patients with screen-detected cancer were significantly higher than those of the patients with interval cancer (P = 0.045). The all-causes survival rate of the patients with interval cancer in the radiographic screening group was not significantly different from that of the patients in the outpatient group (P = 0.771).

The results of the Cox proportional hazards analysis of gastric cancer death and all-causes death in the endoscopic screening group, radiographic screening group, and outpatient group are shown in **Table 3**. Compared with the risk of the outpatient group for gastric cancer death, the hazard ratio of interval cancer in the endoscopic screening group was lower (0.216, 95%CI: 0.054–0.868), but that of interval cancer in the radiographic screening group was equal (1.020, 95%CI: 0.506–2.055). There were no differences among sex, age group, and city in which the patients lived. For all-causes death, although the hazard ratio of the interval cancer in the endoscopic screening group was lower, it was not significantly different (0.420, 95%CI: 0.174–1.014).

**Table 3:** Cox proportional hazard analysis of gastric cancer death and all-causes death in the endoscopic screening group, radiographic screening group, and outpatient group.

	Gastric cancer death			All-causes death		
Characteristics	HR	(95%CI)	P-value	HR	(95%CI)	P-value
**Group**						
**Outpatient group**	**1**	**-**	**-**	**1**	**-**	**-**
**Screen-detected cancer by endoscopic screening**	**0.245**	**(0.171–0.350)**	**< 0.001**	**0.385**	**(0.297–0.497)**	**< 0.001**
**Interval cancer in endoscopic screening**	**0.216**	**(0.054–0.868)**	**0.031**	**0.420**	**(0.174–1.014)**	**0.054**
**Screen-detected cancer by radiographic screening**	**0.368**	**(0.236–0.571)**	**< 0.001**	**0.647**	**(0.481–0.870)**	**0.004**
**Interval cancer in radiographic screening**	**1.020**	**(0.506–2.055)**	**0.957**	**1.104**	**(0.607–2.008)**	**0.746**
**Sex**						
**Male**	**1**	**-**	**-**	**1**	**-**	**-**
**Female**	**0.961**	**(0.776–1.191)**	**0.718**	**0.772**	**(0.640–0.932)**	**0.007**
**Age**						
**40–49 years**	**1**	**-**	**-**	**1**	**-**	**-**
**50–59 years**	**1.109**	**(0.699–1.759)**	**0.660**	**1.121**	**(0.732–1.717)**	**0.600**
**60–69 years**	**1.230**	**(0.793–1.907)**	**0.355**	**1.385**	**(0.926–2.070)**	**0.113**
**70–79 years**	**1.346**	**(0.879–2.060)**	**0.172**	**1.902**	**(1.291–2.804)**	**0.001**
**City**						
**Tottori**	**1**	**-**	**-**	**1**	**-**	**-**
**Yonago**	**0.881**	**(0.702–1.105)**	**0.273**	**0.975**	**(0.810–1.175)**	**0.794**
**Kurayoshi**	**1.154**	**(0.841–1.585)**	**0.374**	**1.133**	**(0.856–1.501)**	**0.383**
**Sakaiminato**	**0.732**	**(0.484–1.110)**	**0.142**	**0.743**	**(0.523–1.056)**	**0.098**

### HR, hazard ratio; CI, confidence interval

The risk factors associated with gastric cancer-specific death and all-causes death in the endoscopic screening group and radiographic screening group were also analyzed (**Table 4**). For gastric cancer death, the hazard ratio of interval cancer in the endoscopic screening group was nearly equal to that of screen-detected cancer in the endoscopic screening group (0.886, 95%CI: 0.213–3.691). Although the hazard ratio of screen-detected cancer in the radiographic screening group was 1.506, it was not significantly different (95%CI: 0.871–2.603). The hazard ratios of interval cancer in the radiographic screening group were always significantly higher: 4.352 for gastric cancer death (95%CI; 2.009–9.427) and 3.091 for all-causes death (95%CI: 1.634–5.849). In the endoscopic screening group, since the hazard ratio of interval cancer was 0.886 for gastric cancer death (95%CI: 0.213–3.691) and 1.117 for all-causes death (95%CI: 0.450–2.771), the risk of interval cancer was nearly equal to that of screen-detected cancer.

**Table 4:** Cox proportional hazard analysis of gastric cancer death and all-causes death for the endoscopic screening group and radiographic screening group.

	Gastric cancer death			All-causes death		
Characteristics	HR	(95%CI)	P-value	HR	(95%CI)	P-value
**Group**						
**Screen-detected cancerby endoscopic screening**	**1**	**-**	**-**	**1**	**-**	**-**
**Interval cancerin endoscopic screening**	**0.886**	**(0.213–3.691)**	**0.868**	**1.117**	**(0.450–2.771)**	**0.811**
**Screen-detected cancer by radiographic screening**	**1.506**	**(0.871–2.603)**	**0.143**	**1.642**	**(1.136–2.373)**	**0.008**
**Interval cancer in radiographic screening**	**4.352**	**(2.009–9.427)**	**< 0.001**	**3.091**	**(1.634–5.849)**	**0.001**
**Sex**						
**Male**	**1**	**-**	**-**	**1**	**-**	**-**
**Female**	**0.786**	**(0.467–1.325)**	**0.367**	**0.465**	**(0.311–0.695)**	**<0.001**
**Age**						
**40–49 years**	**1**	**-**	**-**	**1**	**-**	**-**
**50–59 years**	**1.718**	**(0.211–13.969)**	**0.613**	**2.001**	**(0.250–16.014)**	**0.513**
**60–69 years**	**0.964**	**(0.128–7.247)**	**0.972**	**1.771**	**(0.242–12.979)**	**0.574**
**70–79 years**	**1.333**	**(0.183–9.707)**	**0.776**	**3.249**	**(0.453–23.321)**	**0.241**
**City**						
**Tottori**	**1**	**-**	**-**	**1**	**-**	**-**
**Yonago**	**1.208**	**(0.722–2.022)**	**0.472**	**1.188**	**(0.832–1.695)**	**0.343**
**Kurayoshi**	**0.423**	**(0.058–3.105)**	**0.397**	**0.512**	**(0.125–2.098)**	**0.352**
**Sakaiminato**	**0.757**	**(0.294–1.951)**	**0.564**	**0.663**	**(0.330–1.333)**	**0.249**
**Location**						
**U**	**1**	**-**	**-**	**1**	**-**	**-**
**M**	**0.237**	**(0.130–0.430)**	**< 0.001**	**0.390**	**(0.259–0.588)**	**<0.001**
**L**	**0.338**	**(0.184–0.620)**	**< 0.001**	**0.413**	**(0.264–0.656)**	**<0.001**
**Unknown**	**0.532**	**(0.127–2.238)**	**0.389**	**0.944**	**(0.402–2.219)**	**0.895**
**Stage**						
**Ⅰ**	**1**	**-**	**-**	**1**	**-**	**-**
**Ⅱ**	**7.343**	**(2.831–19.045)**	**< 0.001**	**2.458**	**(1.330–4.543)**	**0.004**
**Ⅲ**	**13.154**	**(5.539–31.237)**	**< 0.001**	**3.197**	**(1.789–5.712)**	**< 0.001**
**IV**	**52.876**	**(20.820–134.284)**	**< 0.001**	**12.244**	**(5.967–25.124)**	**< 0.001**
**Unknown**	**4.881**	**(2.287–10.418)**	**< 0.001**	**1.760**	**(1.179–2.626)**	**0.006**
**Histology**						
**Intestinal type**	**1**	**-**	**-**	**1**	**-**	**-**
**Diffuse type**	**3.403**	**(2.028–5.711)**	**< 0.001**	**1.639**	**(1.134–2.367)**	**0.009**
**Others**	**2.663**	**(0.361–19.639)**	**0.337**	**2.964**	**(0.934–9.404)**	**0.065**
**Unknown**	**3.956**	**(1.518–10.310)**	**0.005**	**2.179**	**(1.051–4.515)**	**0.036**

Group

HR, hazard ratio; CI, confidence interval

## Discussion

The present study showing the survival rate of patients with interval cancer indicated that the endoscopic screening group had a better prognosis than the radiographic screening group and outpatient group, as demonstrated by the results of gastric cancer-specific survival and all-causes survival analyses. The survival rate and the risk of gastric cancer death for patients with interval cancer were similar to those of patients with screen-detected cancer in the endoscopic screening group. Thus, interval cancer can potentially be used as an indicator for predicting the early effects of cancer screening. Interval cancer includes cases missed at the previous screening and cases which appeared because they grew rapidly as the preclinical phase (sojourn time) was shorter than the screening interval [15, 16]. Because of the good prognosis of interval cancer in endoscopic screening, the results suggest a possibility of reducing mortality from gastric cancer by endoscopic screening. However, this can be misleading because the survival rate of patients with screen-detected cancers is overestimated by length bias, lead time bias and overdiagnosis. Since we used the survival rate of patients with screen-detected cancers for comparison, there is a need for prudent interpretation of the survival rate of patients with interval cancer in the present study.

On the other hand, sensitivity can also be a factor for predicting the effectiveness of cancer screening. Greater sensitivity leads to high cancer detection rates during screening and lower interval cancer rates. Several studies have reported that the sensitivity of endoscopic screening is usually higher than that of radiographic screening [7, 8]. This implies that the rate of interval cancer is lower in endoscopic screening than in radiographic screening. Since endoscopic screening has a potential to detect early-stage cancer, localized cancer was reportedly more frequent in patients who had undergone endoscopic screening than in those who had undergone radiographic screening [8, 17, 18]. In mammographic screening, several studies have shown that interval cancers and screen-detected cancers have different clinicpathologic characteristics [15, 16, 19–21]. Although we could not obtain detailed information regarding the specific clinical stage of the interval cancers, the interval cancers on endoscopic screening for gastric cancer in a previous study were early-stage cancers only, whereas those on radiographic screening included late-stage cancers [7].

The survival rates of patients with interval cancer have been reported to be lower than those of patients with screen–detected cancer in mammographic screening [19, 20]. In the present study involving endoscopic screening for gastric cancer, the survival rates of the patients with screen-detected cancer and the patients with interval cancers were not significantly different and higher than that of patients in the outpatient group. The risk of gastric cancer death from interval cancer in the endoscopic screening group was similar to that of gastric cancer death from screen-detected cancer in the endoscopic screening group. Although the screening interval was 1 year for endoscopic screening and radiographic screening in the study areas, a better prognosis might be expected for endoscopic screening. These results suggest that it may be possible to extend the endoscopic screening interval to more than 1 year. In fact, mortally reduction was shown in the screening programs in Korea with a screening interval of 2 years [8]. Although the number of endoscopic examinations has rapidly increased in Japan [22], insufficient capacity may be more of a barrier for endoscopic screening not to be introduced in local communities. If the screening interval can be extended, endoscopic screening may be used efficiently even with limited resources.

A notable constraint of the present study is the lack of data regarding the clinical stage of the interval cancer. To evaluate the effects of interval cancer, follow-up of the participants of a population-based screening based on the cancer registry is needed. In Japan, cancer registries have not yet been prepared at the national level, and the registry method has not yet been standardized as of 2014 [23, 24]. The Tottori Cancer Registry is one of the most reliable systems with a long history in Japan. Although information about disease extension has been obtained as an alternative item for the clinical stage, this information is often lacking [25]. The quality of the Tottori Cancer Registry was, however, not optimal since the percentage of death-certification-only cases was 15.1% in 2007 which was lower than the national average [26]. Even if there was a notification of new cases in the cancer registration system, detailed information was often lacking because the clinical stage was not a necessary item. Fortunately, additional information could be obtained for the screening group from the Tottori Medical Association database because the association has the responsibility of implementing gastric cancer screening programs and collecting detailed information for quality assurance. However, we could not obtain additional detailed information regarding the numbers of medical institutions in Tottori Prefecture for the outpatient group and the interval cancer cases in both screening groups. These limitations prevented us from obtaining stage information sufficiently, thus careful interpretation of the results in reference to these contains is required.

This study has other limitations. First, the background difference should be considered between the endoscopic screening group and the radiographic screening group. Endoscopic screening has been performed in clinical practice in Tottori Prefecture. The age of the participants in endoscopic screening was more advanced than that of the participants in radiographic screening [7]. Individuals aged more than 70 years could be screened by physicians using endoscopy in their own private practice. Since younger people who have family physicians were fewer than older people who have family physicians, there was little opportunity for the younger people to be tested in clinical practice. Second, since there was no information as to whether or not the patients participated in opportunistic screenings, the outpatient group might include cancer patients which were detected by these screenings. Selection bias may also be considered in the selection of the screening method at the individual level. Third, the survival rate was different among hospitals in Japan [27]. Moreover, the present results are limited to local areas in Japan. Finally, subgroup analysis could not be adequately performed because of the small sample size.

In conclusion, the gastric cancer-specific and all-causes survival rates of patients with screen-detected cancers and patients with interval cancers were nearly equal in the annual endoscopic screening. The risk of gastric cancer death was lower in the patients with screen-detected and interval cancers in the endoscopic screening group than in the outpatient group. These results suggest the potential of endoscopic screening in reducing mortality from gastric cancer. However, additional studies must be performed to more extensively evaluate mortality reduction from gastric cancer by endoscopic screening as well as to investigate the impact of interval cancer on the effectiveness of endoscopic screening for gastric cancer.
